# Complete Genome Sequences of Four Staphylococcus aureus Sequence Type 398 Isolates from Four Goats with Osteomyelitis

**DOI:** 10.1128/MRA.01174-18

**Published:** 2018-09-27

**Authors:** Mohamed A. Abouelkhair, Rebecca E. Rifkin, Remiqiusz M. Grzeskowiak, Alexandru S. Biris, David E. Anderson, David A. Bemis, Stephen A. Kania

**Affiliations:** a Department of Biomedical and Diagnostic Sciences, College of Veterinary Medicine, University of Tennessee, Knoxville, Tennessee, USA; b Department of Large Animal Clinical Sciences, College of Veterinary Medicine, University of Tennessee, Knoxville, Tennessee, USA; c Center for Integrative Nanotechnology Sciences, University of Arkansas at Little Rock, Little Rock, Arkansas, USA; Loyola University Chicago

## Abstract

Staphylococcus aureus is the causative agent of multiple infections, including bacteremia, infective endocarditis, osteomyelitis, septic arthritis, and prosthetic device infections. We report here the first whole-genome sequence for four S. aureus sequence type 398 isolates from clinical cases of osteomyelitis in four goats with a history of orthopedic surgery.

## ANNOUNCEMENT

Staphylococcus aureus is a Gram-positive bacterium commonly found as a component of the human and animal normal flora (1–3). S. aureus is an important opportunistic pathogen in human medicine and is an emerging problem in veterinary practice (1, 2, 4).

Here, we report draft genome sequences of four methicillin-susceptible S. aureus (MSSA) sequence type 398 (ST398) isolates obtained from goats that underwent orthopedic surgery at the College of Veterinary Medicine, University of Tennessee (Table 1). The goats were 5 to 6 years old, weighed between 41 and 61 kg, and were free of underlying disease at the time of surgery. The goats had undergone orthopedic surgery a mean of 45 days prior (range, 30 to 90 days) to clinical manifestation of osteomyelitis. Clinical signs included nonhealing wounds, deep surgical site infections, and prolific bone formation. This work was approved by the University of Tennessee IACUC and conducted under protocol 2383-1215.

**TABLE 1:** Strain characteristics

Characteristic	Data for isolate:			
	UTCVM 1	UTCVM 2	UTCVM 3	UTCVM 4
Laboratory accession no.	MI18-33	MI18-34	MI18-935	MI18-1974
GenBank accession no.	QQZR00000000	QQZS00000000	QQZU00000000	QQZX00000000
No. of reads	552,318	760,362	735,277	920,750
No. of contigs	29	28	27	28
*N*_50_ (bp)	193,883	227,498	380,234	228,010
Genome length (bp)	2,835,200	2,834,945	2,835,359	2,836,056
G+C content (%)	32.9	32.9	32.9	32.9
No. of predicted coding sequences	2,793	2,793	2,793	2,790
No. of predicted RNAs	76	75	77	78
SRA accession no.	SRR7765197	SRR7765198	SRR7765195	SRR7765196

A single bacterial colony of each strain grown on blood agar plates was inoculated into 5 ml of sterile Trypticase soy broth (TSB) (BD Biosciences, USA; cat. no. RS1-011-21) and incubated overnight at 37°C with shaking at 225 rpm using an Excella E24 incubator shaker (New Brunswick Scientific, USA). DNA was extracted using the MasterPure DNA purification kit (Epicentre, USA; cat. no. MCD85201) according to the manufacturer's instructions.

Sequencing libraries were constructed using the Nextera DNA sample prep kit (Illumina, Inc., USA) according to the manufacturer's instructions. The genomes were sequenced using a MiSeq platform (Illumina, Inc.) with a single-end read length of 150 bp at the University of Tennessee Genomics Core facility. Sequences were trimmed using BBDuk and *de novo* assembled using Geneious version 11.0.3 (5). Annotation was performed by the NCBI Prokaryotic Genome Annotation Pipeline version 4.6 (https://www.ncbi.nlm.nih.gov/genome/annotation_prok) using the best-placed reference protein set with GenMarkS+. The numbers of reads and contigs, *N*_50_ values, G+C content values, and the total lengths of the draft genome sequences are listed in (Table 1).

In the genome sequence of S. aureus strain MI18-33, there are 2,793 putative protein-coding genes out of 2,905 predicted genes with 112 pseudogenes. Strain MI18-34 contained 2,793 putative protein-coding genes and 109 pseudogenes out of 2,902 predicted genes. Strain MI18-935 had 2,793 putative protein-coding genes out of 2,904 predicted genes with 111 pseudogenes. In strain MI18-1974, there are 2,790 putative protein-coding genes and 111 pseudogenes out of 2,901 predicted genes.

This work used the S. aureus MLST database at the University of Oxford (https://pubmlst.org/saureus) for whole-genome multilocus sequence typing (6). All isolates were identified as ST398.

The genome sequences will facilitate identification of the genetic relatedness of these isolates with previously published S. aureus strains and may help to elucidate a pathomechansim involved in bone infections, healing, and regeneration (7–9).

### Data availability.

The whole-genome shotgun sequencing projects of isolates S. aureus MI18-33 (UTCVM 1), MI18-34 (UTCVM 2), MI18-935 (UTCVM 3), and MI18-1974 (UTCVM 4) have been deposited at DDBJ/ENA/GenBank and the Sequence Read Archive (SRA) under the accession numbers listed in Table 1.
